# Osteoporosis: a missed link in fall prevention - a retrospective cohort study

**DOI:** 10.1186/s12877-026-07119-0

**Published:** 2026-02-19

**Authors:** Nadja Fries, Agathi Constantinou, Therése Wretborn, Anna Spångeus

**Affiliations:** 1 Division of Diagnostics and Specialist Medicine, Department of Health, Medicine and Caring Sciences, Campus US, Linköping University, Linköping, 581 83 Sweden; 2 Department of Activity and Health, Linköping University Hospital, Linköping, Sweden; 3 Department of Geriatrics and Palliative Medicine, Linköping University Hospital, Linköping, Sweden

**Keywords:** Osteoporosis, Fall, Fracture, Geriatric, Risk assessment

## Abstract

**Background:**

Falls and osteoporotic fractures are common in the geriatric population. Although falls increase the risk of osteoporotic fractures, many patients with recurrent falls are untreated for osteoporosis. In our study, we aimed to; (1) assess the incidence of fractures among patients who experience falls during inpatient care in a geriatric ward; (2) investigate the risk factors associated with fractures in this cohort; and (3) investigate whether patients on anti-osteoporotic treatment differed from and had better fall-related clinical outcomes compared to those without anti-osteoporotic treatment.

**Methods:**

This study involved a retrospective cohort analysis of inpatient falls in the Geriatric ward between 2018 and 2020. Patients who experienced a fall during this period were identified through the hospital’s adverse event tracking system. Demographic and clinical data, fall-related information, associated risk factors and outcomes were extracted from the hospital’s electronic medical records.

**Results:**

A total of 159 patients with fall incidents were included, with the mean age of the patients being 84 ± 7 years, and 45% of them being female. Among these patients, 9% sustained a fracture during the fall, with hip and vertebral fractures being the most common types. Nearly half of the patients who had fallen (42%) had experienced at least one previous fracture. None of the patients who sustained a fracture during inpatient care were currently receiving anti-osteoporotic treatment, compared to 8% in the non-fracture group. Most patients (97%) were considered to have a high risk of falling (Downton fall risk index *≥* 3).

**Conclusions:**

Although risk assessments and interventions are conducted to prevent injurious falls, patients continue to experience falls. Despite being classified as high-risk fallers and having a history of previous fractures, only a minority of these patients are receiving anti-osteoporotic treatment. This shortfall could potentially contribute to the high incidence of fall-related fractures observed in this study.

**Graphical Abstract:**

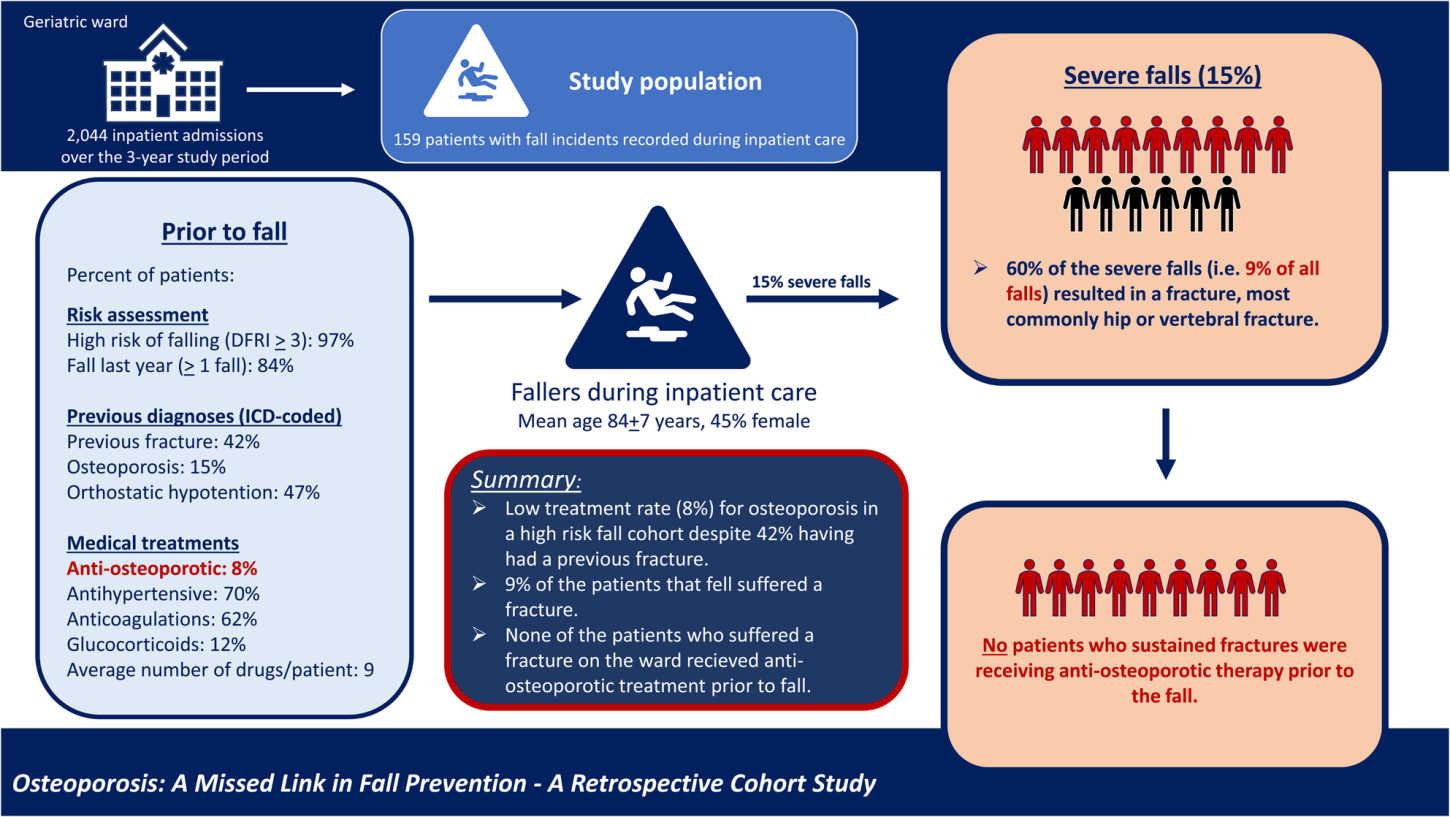

## Key Points

Fractures occurred in 9% of geriatric inpatients with a documented fall, with hip and vertebral fractures being the most common.

None of the patients who sustained fractures were receiving anti-osteoporotic treatment, despite being classified as high-risk fallers.

The underutilization of osteoporosis treatment in this vulnerable population may contribute to the high incidence of fall-related fractures.

## Why does this paper matter?

This study highlights a critical gap in osteoporosis management among high-risk geriatric inpatients, emphasizing the need for improved treatment strategies to prevent fall-related fractures and enhance patient outcomes.

## Background

Osteoporosis is a common skeletal disease characterized by reduced bone mass and deterioration of bone microarchitecture, resulting in diminished bone strength and an increased risk of fragility fractures. This condition is particularly common in the aging population, as bone mineral density (BMD) declines with age [1]. With rising life expectancy, the prevalence of fragility fractures is expected to rise [2]. These fractures are associated with substantial morbidity, including persistent disability and impaired quality of life, as well as considerable health care costs (such as hospitalization), and increased mortality rates [3–6]. Importantly, the risk of sustaining a subsequent fracture is significantly higher in the years following an initial fracture [7] underscoring the importance of preventive strategies.

Falls are frequent among older hospitalized individuals and represent a major risk factor for fracture [6, 8]. Multiple comorbidities, polypharmacy and the use of certain medications, such as sedatives and antihypertensives, further increase fall risk in older adults [9]. However, the clinical need for these treatments often escalates with age, highlighting the necessity of individualized, carefully balanced risk–benefit assessments.

To mitigate hospital falls, multifactorial interventions are recommended, including patient and staff education, systematic use of fall-risk assessment tools, regular medication reviews, tailored physical exercise programs, and environmental modification [9–11].

Despite the strong link between falls and fractures, and the availability of well-documented and effective fracture prevention treatments [12–15], only a minority of patients with osteoporosis are identified and treated [2, 16]. Therefore, integrating osteoporosis assessment into fall and fall-injury prevention strategies is essential [9]. Many clinical guidelines emphasize fall prevention rather than fall-injury prevention, the latter encompassing measures to enhance endogenous resistance to fall injuries such as optimizing skeletal health, despite a clear treatment gap among high-risk fallers.

In the present study, we aimed to; (1) assess the incidence of fractures among patients who experience falls during inpatient care in a geriatric ward; (2) investigate the risk factors associated with fractures in this cohort; and (3) investigate whether patients on anti-osteoporotic treatment differed from and had better fall-related clinical outcomes compared to those without anti-osteoporotic treatment.

## Methods

### Study cohort and variables

In this retrospective study, patients who experienced a fall during inpatient care in the geriatric ward at Linköping University Hospital in Sweden over a three-year period (January 1, 2018, to December 31, 2020) were included. Information regarding the falls, associated risk factors, and outcomes was extracted from the hospital’s electronic medical records. Fall incidents were identified by the hospital’s adverse event tracking system (Synergi) and were defined in accordance with the definition outlined by the World Health Organization as an event which results in a person coming to rest inadvertently on the ground or floor or other lower level [17]. Exclusion criteria included fall incidents secondary to clinical events (e.g., stroke, seizure, arrhythmia). A fall was considered severe if it resulted in a fracture, the need for opioid prescription, major head injury, internal bleeding, or death.

Different risk assessments were routinely evaluated for all patients upon admission to the ward and were documented in the medical records. These included Elderly Mobility Scale (M-EMS), Downton Fall Risk Index (DFRI), nutrition status (Short Form Mini Nutritional Assessment, SF-MNA), and risk of developing pressure sore (Risk Assessment Pressure Sore, RAPS) [18–21].

Current medications were reviewed from the medical records and defined as those prescribed (i.e. listed on medication chart) to patients within 24 h prior the fall, except for Zoledronic acid, which was included if administered up to one year prior to the fall and denosumab if administered up to 6 months prior to the fall. For analysis purposes medications were divided into sub-groups i.e.; (1) psychotropics including sedatives-hypnotics and antipsychotic medications; (2) antihypertensives including diuretics, beta-blockers, renin-angiotensin system (RAS) agents, alpha-1 antagonists, and calcium channel blockers; and (3) antithrombotics, including anticoagulants and platelet inhibitors. In addition, a variable containing the number of medications classified as fall risk increasing drugs (FRIDs) was included [22].

Previous fractures were identified in two ways; (1) if ICD-coded (International Statistical Classification of Diseases and Related Health Problems) in the medical record; or (2) if ICD-coded and/or identified having a previously undiagnosed vertebral fractures in opportunistic screening from CT scans performed in near time from fall (data from a sub-study [23]). ICD codes were available in the medical records from 2008.

### Statistical analysis

Continuous variables were presented as mean and standard deviation (SD) or median and 25–75 percentile, depending on their normality. Categorical variables were expressed as frequencies. Group comparisons for continuous data were performed using either the T-test or the Mann-Whitney U test, based on normality, while the Chi-square test was used for categorical data. Data analysis was conducted using IBM SPSS (version 29.0.2.0, IBM Corp., Armonk, NY, USA). A p-value of < 0.05 was considered statistically significant.

### Ethics

The study protocol was reviewed and approved by the Swedish Ethical Review Authority (2021 − 01425, with an amendment 2022-02053-02). Informed consent was not required for this retrospective cohort study design.

## Results

### Study population and incidence of fractures

**Fig. 1:**
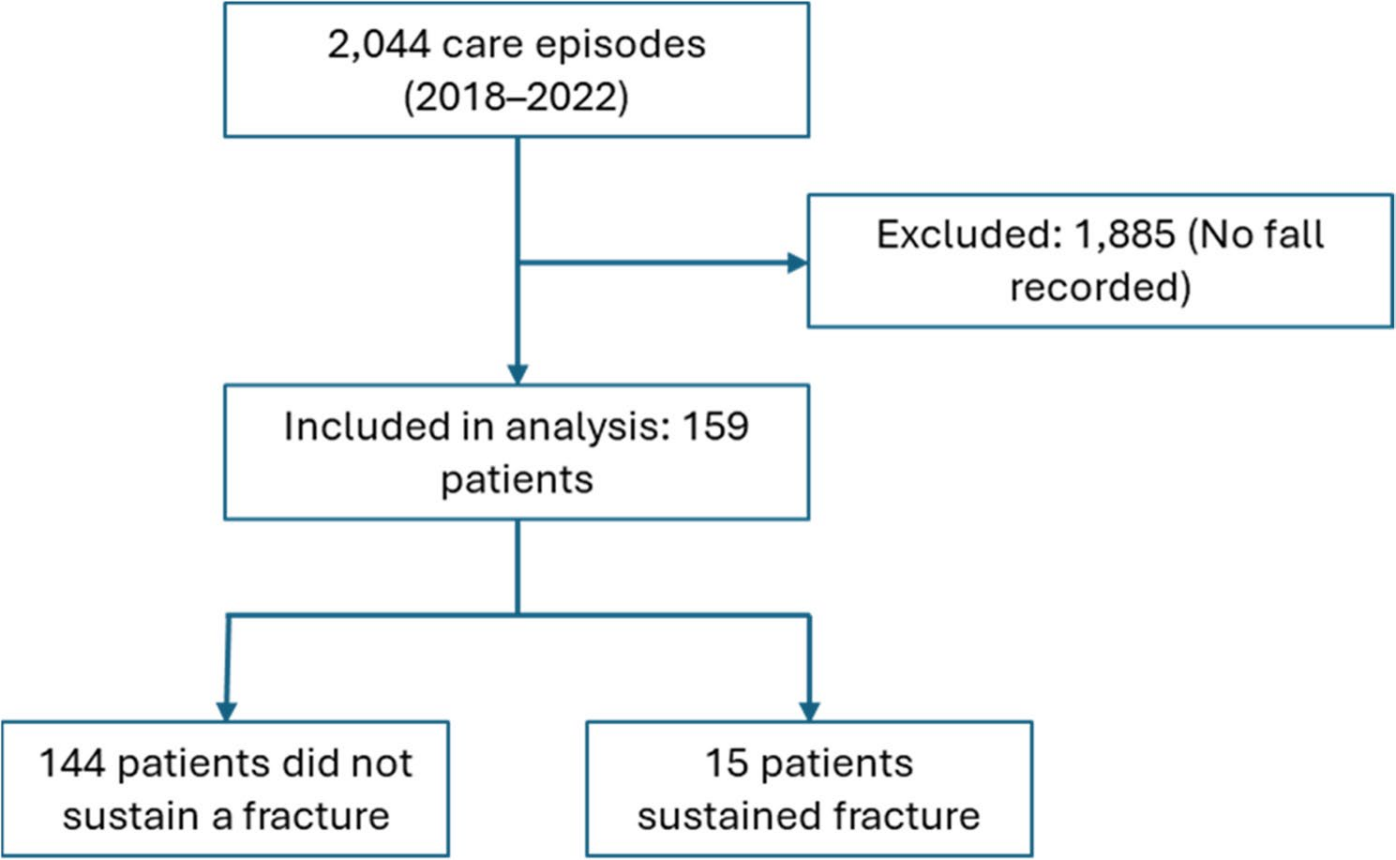
Flow chart describing the study cohort

**Fig. 2:**
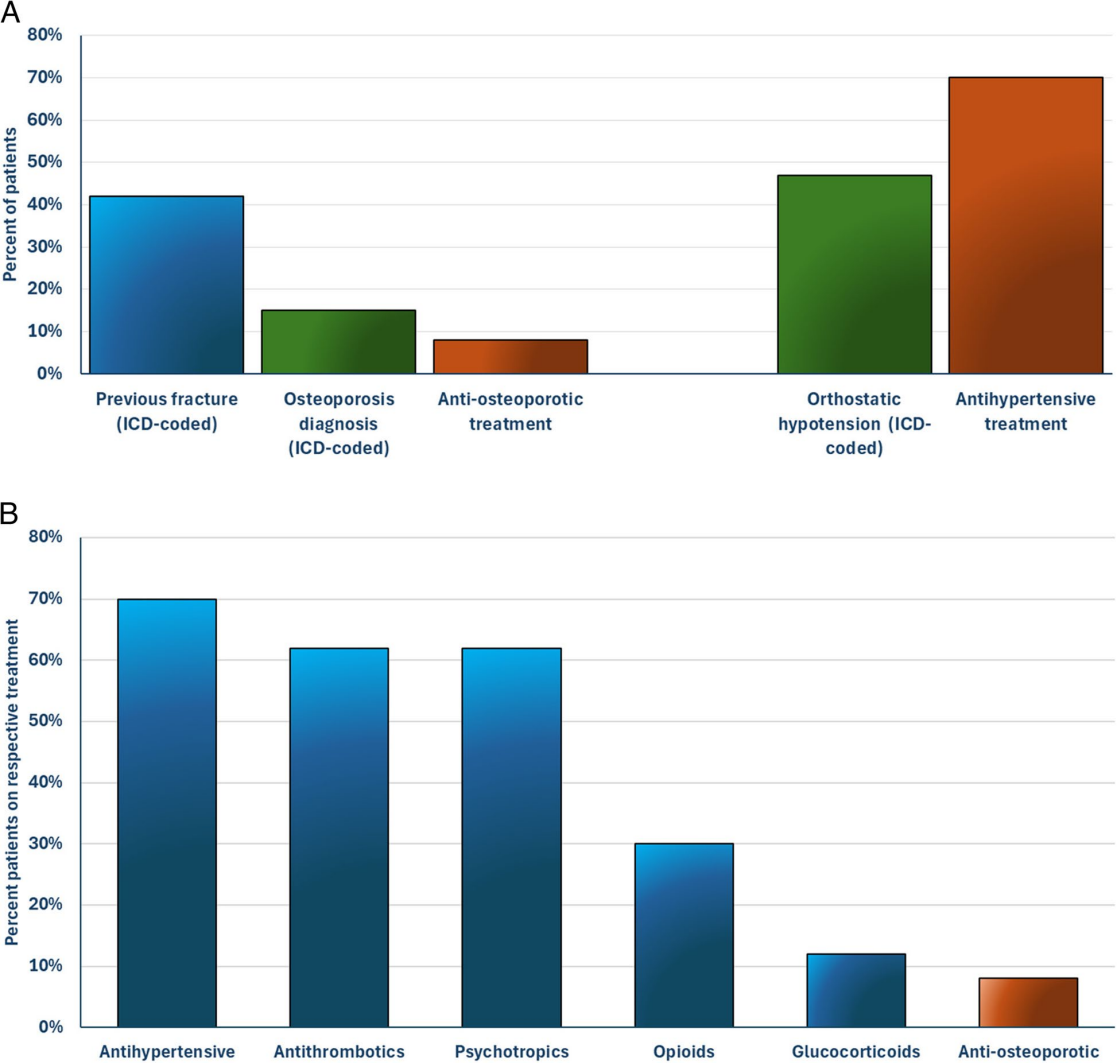
(**a**) The proportion of patients diagnosed with prior fractures, osteoporosis, and/or orthostatic hypotension, compared to the proportion of patients receiving anti-osteoporotic and/or antihypertensive treatment. (**b**) Patients on respective treatment prior to the fall. Antihypertensives: diuretics, beta-blockers, renin-angiotensin system (RAS) agents, alpha-1 antagonists, or calcium channel blockers; Antithrombotics: anticoagulants and platelet inhibitors; Psychotropics: sedatives-hypnotics and antipsychotic medications

Descriptive statistics are summarized in Table 1. Throughout the 3-year study period, the care unit had 2,044 care episodes. A total of 159 patients (mean age 84 years, 45% females) experienced a documented fall incident during their inpatient stay, thus falls occurred in 8% of all care episodes at the ward. Of the 159 patients with a fall, 23 falls were classified as severe and included 15 patients who sustained fractures (Fig. 1). The most common type of fracture was hip fractures (*n* = 5) followed by vertebral fractures (*n* = 4). Other types of fractures included rib, clavicle, facial, and humerus fractures. Among the 159 patients, 42% had a history of at least one ICD-coded fracture (Fig. 2a). When including non-ICD-coded vertebral fractures identified through opportunistic CT screening, the percentage increased to 52%. Despite the high prevalence of previous fractures in this cohort, only a small proportion of patients (15%) were diagnosed with osteoporosis.

**Table 1:** Demographic and clinical characteristics of patients

	Variable	All (*n* = 159)	Missing
Demographics	Age	84 ± 7	0
	Sex, female	45%	0
	BMI	24 [21–27]	12
Assessment Score	DFRI	5 [4–6]	24
	DFRI *≥* 3	97%	
	M-EMS	10 [6–14]	10
	SF-MNA	8 [6–10]	25
	SF-MNA *≤* 11	93%	
	RAPS	30 [28–32]	26
	RAPS < 29	31%	
	Previous fall (*≥* 1) last year	84%	1
Medication	Total number of medications	9 ± 4	0
	Total number of FRID-classified medication	3 [2–5]	0
	Any FRID-classified medication	91%	0
Medical History*	Osteoporosis	15%	0
	Previous fracture	42%	0
	Orthostatic hypotension	47%	0
	Cognitive disease	34%	0
	Parkinson disease	14%	0
Fall incident	Fall-related fracture	9%	0
	Severe fall	15%	0
	Length of stay	16 [10–27]	0
	30-day mortality	16%	0
	One year mortality	45%	0

Values presented as mean ± SD, median [25 - 75 percentile] or percent. *ICD-coded. *BMI* Body Mass Index, *FRID* Fall-Risk Increasing Drugs, *DFRI* Downton Fall Risk Index, *M-EMS* Modified Elderly Mobility Scale, *SF-MNA* Short form Mini Nutrition Assessment, *RAPS* Risk Assessment Pressure Sore scale

Nearly all participants were considered as high risk of falling according to the DFRI, with moderate mobility limitations, averaging 10 points on the M-EMS. This aligns with the fact that 84% of participants were recurrent fallers (*≥* 1 fall in the past year). As a result of high DFRI scores, fall prevention measures were implemented and documented in the medical records in 69% of patients during their inpatient care. Orthostatic blood pressure was evaluated in 73 participants during their inpatient stay, with 64% showing a pathological reaction (orthostatic hypotension). Additionally, 47% of all patients had ICD-coded orthostatic hypotension prior to admission (Fig. 2a).

As shown in Fig. 2b, over half of the participants were receiving antihypertensives, antithrombotics, and/or psychotropics (70%, 62%, and 62%, respectively). Nearly one-third of the participants were receiving opioids (30%), and 12% glucocorticoids. In contrast, only 8% were receiving anti-osteoporotic treatment. On average, patients were prescribed 9 medications and a total of 91% of the patients were prescribed at least one FRID-classified medication.

### Fracture vs. no fracture related to the fall

Risk factors for patients who sustained a fracture compared to those who did not are summarized in Table 2. Most fractures occurred in males, i.e. 11 males compared to 4 females. No significant age difference was observed between patients with or without fractures. There was a trend towards lower BMI in patients who sustained a fracture during the fall (BMI 22 [21–24] vs. 24 [20–27] kg/m², *p* = 0.177). Significantly fewer patients with a high BMI (*≥* 25 kg/m²) sustained a fracture after the fall falls (2%) than patients with normal weight (15%, *p* = 0.015). No statistically significant difference was seen between patients with underweight (< 18.5 kg/m²) and normal weight (6% vs. 15%, *p* = 0.453).

The prevalence of previous fractures was high in both groups (42–47%). Despite this, only a minority of the patients had an osteoporosis diagnosis (7–15%). Though non-significant, the diagnosis of osteoporosis was twice as common in the non-fracture group compared to the fracture group (15% vs. 7%). Similarly, 8% of the patients in the non-fracture group were prescribed anti-osteoporotic treatment prior to the fall, in comparison to none of the patients in the fracture group. Conversely, glucocorticoid treatment was nearly twice as common in the fracture group compared to the non-fracture group (20% vs. 11%). The length of hospitalization was longer, though statistically non-significant, in the fracture-group compared to the non-fracture group (median 19 days and 16 days respectively). No statistical difference was observed between the groups regarding risk scores or fall preventive measures.

**Table 2:** Comparison between the fracture vs. non-fracture patient groups related to the fall

	Variable	Fracture (*n* = 15)	No fracture (*n* = 144)	*p*-value
Demographics	Age	86 ± 6	84 ± 7	0.479
	Sex, female	27%	47%	0.141
	BMI (kg/m^2^)	22 [21–24]	24 [20–27]	0.177
Assessment Score	DFRI	5 [4–6]	5 [4–6]	0.531
	DFRI *≥* 3	100%	98%	0.524
	M-EMS	9 [6–14]	10 [6–14]	0.907
	SF-MNA	7 [4–8]	8 [6–10]	0.128
	SF-MNA *≤* 11	100%	92%	0.326
	RAPS	29 [25–31]	31 [28–32]	0.181
	RAPS < 29	36%	30%	0.678
	Previous fall (*≥* 1) last year	93%	83%	0.307
Medication	Opioids	27%	30%	0.796
	Psychotropics	60%	62%	0.891
	Antithrombotics	87%	60%	0.040
	Antihypertensives	73%	69%	0.755
	Glucocorticoids	20%	11%	0.312
	Anti-osteoporotic	0%	8%	0.245
	FRID-classified medication	100%	90%	0.189
	Total number of FRID-classified medication	3 [2–5]	3 [2–5]	0.510
	Total number of medications	9 ± 3	9 ± 4	0.841
Medical History*	Osteoporosis	7%	15%	0.367
	Previous fracture	47%	42%	0.709
	Orthostatic hypotension	33%	48%	0.281
	Cognitive disease	47%	33%	0.275
	Parkinson disease	27%	13%	0.133
Fall incident	Length of stay	19 [12–26]	16 [10–27]	0.704
	30-day mortality	20%	15%	0.633
	One year mortality	40%	46%	0.666

Values presented as mean ± SD, median [25 - 75 percentile] or percent. *ICD-coded. *BMI* Body Mass Index, *FRID* Fall-Risk Increasing Drugs, *DFRI* Downton Fall Risk Index, *M-EMS* Modified Elderly Mobility Scale, *SF-MNA* Short form Mini Nutrition Assessment, *RAPS *Risk Assessment Pressure Sore scale

### Anti-osteoporotic treatment vs. no anti-osteoporotic treatment

A summary of patients with anti-osteoporotic treatment vs. no anti-osteoporotic treatment is shown in Table 3. Overall, the proportion of patients receiving treatment for osteoporosis was low, i.e. 8%. There was no significant difference regarding age and sex between patients treated or not treated with anti-osteoporotic drugs.

Patients with ongoing anti-osteoporotic treatment were more frequently diagnosed with osteoporosis and had a higher total number of medications prescribed. Furthermore, the patients with anti-osteoporotic treatment showed a trend (*p* = 0.074) towards having experienced a previous fracture more frequently.

**Table 3:** Comparison of patients with anti-osteoporotic treatment vs. no anti-osteoporotic treatment

	Variable	Anti-osteoporotic treatment (*n* = 12)	No anti-osteoporotic treatment (*n* = 147)	*p*-value
Demographics	Age	86 ± 6	84 ± 7	0.430
	Sex, female	58%	42%	0.322
	BMI (kg/m^2^)	24 [20–27]	24 [21–27]	0.914
Assessment Score	DFRI	5 [3–7]	5 [4–6]	0.442
	DFRI *≥* 3	100%	97%	0.586
	M-EMS	7 [6–10]	10 [6–14]	0.384
	SF-MNA	8 [4–10]	8 [6–10]	0.792
	SF-MNA *≤* 11	100%	92%	0.351
	RAPS	31 [25–32]	30 [28–32]	0.770
	RAPS < 29	33%	31%	0.866
	Previous fall (*≥* 1) last year	91%	84%	0.526
Medication	Opioids	17%	31%	0.309
	Psychotropics	67%	61%	0.709
	Antithrombotics	83%	61%	0.117
	Antihypertensives	83%	69%	0.289
	Glucocorticoid	25%	11%	0.147
	FRID-classified medication	100%	90%	0.245
	Total number of FRID-classified medication	5 [4–6]	3 [2–4]	0.009
	Total number of medications	13 ± 2	9 ± 4	< 0.001
Medical History*	Osteoporosis	83%	9%	< 0.001
	Previous fracture	67%	40%	0.074
	Orthostatic hypotension	58%	46%	0.394
	Cognitive disease	42%	33%	0.558
	Parkinson disease	25%	13%	0.220
Fall incident	Length of stay	21 [11–27]	16 [10–27]	0.396
	30-day mortality	8%	16%	0.465
	One year mortality	67%	44%	0.122

Values presented as mean ± SD, median [25 - 75 percentile] or percent. *ICD-coded. *BMI* Body Mass Index, *FRID* Fall-Risk Increasing Drugs, *DFRI* Downton Fall Risk Index, *M-EMS* Modified Elderly Mobility Scale, *SF-MNA* Short form Mini Nutrition Assessment, *RAPS *Risk Assessment Pressure Sore scale

## Discussion

In the present study we show that the incidence of fractures was high during geriatric inpatient care with nearly one in ten patients that had a documented fall during their hospital stay sustaining a fracture related to the fall. Most of these were hip or vertebral fractures. Only 8% of the patients had ongoing anti-osteoporotic treatment; despite half of the patients having had a previous fragility fracture, nearly all (97%) being considered as high-risk for fall (DFRI assessed), and 84% being recurrent fallers (*≥* 1 fall recent year). None of the patients that fractured during the fall on the ward were on anti-osteoporotic treatment prior to the fall.

Fractures related to fall incidents during hospital care have previously been reported from other inpatient settings such as in Australia (3.8%) and Brazil (< 2.1%) [24, 25]. The latter study also included younger patients (average age 65 years) but concluded that fractures were more common after falls in the oldest patients, thus in the age group most similar to our study. If a patient had fallen multiple times our study included the most severe fall, this may thereby partly explain our higher frequency of fractures. Another factor that may explain the higher fracture incidence in our study is a higher mean age in our cohort. Furthermore, the definition of fall incidence differs between studies. Thus, in our study, a fall-incidence was strictly defined as incidents where patients actually fell to ground level or a lower level. In contrast, other studies also included fall incidents where the actual fall was prevented by healthcare professionals assisting the patient to an armchair or the floor [25].

The patients in our study exhibited several strong risk factors for osteoporosis, including advanced age (mean 84 years), high prevalence of previous fractures, glucocorticoid treatment, recurrent falls, high fall risk, and risk of malnutrition. Despite these risk factors, only a minority of patients, i.e. 15%, were diagnosed with osteoporosis, and even fewer (8%) received anti-osteoporotic treatment. These findings indicate that osteoporosis remains significantly underdiagnosed and undertreated in this patient population, underscoring the need to prioritize osteoporosis assessment and management alongside fall prevention strategies in geriatric care. In our cohort, 8% of patients in the non-fracture group had received anti-osteoporotic treatment prior to the fall, whereas none (0%) in the fracture group had been treated. Although this difference appears clinically relevant, it did not reach statistical significance, most likely due to insufficient statistical power. Based on our calculations, approximately twice the number of observations would have been required to detect such a difference with adequate power.

As previously mentioned, it is, however, important to value each patients’ current risk versus benefit when prescribing additional medications, particularly in this fragile patient group. On the other hand, other medical treatments, including FRID medications, were frequently prescribed, i.e. 91% of patients had at least one, and the mean number of medications was considerable. i.e. mean number 9, indicating that this patient group was considered to benefit from active treatments. Thus, it seems unlikely that the non-treatment for osteoporosis was an active decision.

A cohort study conducted in Canada examined anti-osteoporotic treatment in patients (*≥* 65 years) who previously sustained a hip fracture [26]. Similarly to our findings, only 38 of 449 patients (8%) were receiving anti-osteoporotic treatment prior to fracture and 23% were receiving it post-fracture. Mortality post-fracture was significantly lower in the group receiving anti-osteoporotic treatment. In general, osteoporosis treatment rates remain low after fractures, despite clear guidelines and efforts to develop fracture liaison services (FLS) [2]. While a history of previous fracture was similarly high in both the fracture and non-fracture group in our study, osteoporosis diagnoses (M80.x and M81.x) were twice as common in the non-fracture group. Additionally, glucocorticoid use, a well-known risk factor for osteoporosis [27], was nearly twice as common in the fracture group. These findings might suggest a higher degree of underdiagnosis and undertreatment in the fracture group.

Although the overall treatment rate with anti-osteoporotic medications in our study was low, both a prior diagnosis of osteoporosis and a history of previous fragility fractures were associated with a higher likelihood of receiving treatment. The proportion of treated patients was lower than national data on secondary fracture prevention, including younger patients as well (12%) [28]. This is despite previous studies indicating comparable efficacy of anti-osteoporotic treatments in ageing patients as in the younger [29]. In a study by Ek et al., the rate of secondary fracture prevention with anti-osteoporotic therapy was two to three times lower in patients aged 90 years and above compared to those aged 70–89 years [16].

Regarding previous fractures, 42% of the patients had sustained one or more previous fractures, according to ICD coding. In the present fall cohort, radiological examinations of available CT scans were performed in another sub-study, revealing a considerable number of undiagnosed vertebral fractures (i.e. an additional 10% points in the previous fracture sub-group) [23]. The underdiagnosing of vertebral fractures is well known and today less than one-third are diagnosed [30–32]. Including these non-ICD-coded fractures, a total of 52% of patients in our cohort were found to have had a previous fracture. This suggests that fractures in the geriatric population are very common and that vertebral fractures are often missed or overlooked. Given that previous fractures and specifically vertebral fractures, are strong risk factors for osteoporosis and future osteoporotic fractures, it is crucial to improve the diagnosis and treatment rates after these fractures [33].

In our cohort, fall risk and mobility assessments were typically conducted prior to the fall. The results of these assessments correlated well with the clinical outcomes, as 97% of all the patients who fell at our ward were classified as at risk of falling (DFRI *≥* 3). Nevertheless, DFRI was not associated with the severity of the fall, including fractures which seems logical as DFRI aims to identify individuals at high risk of fall, not the outcome of the fall (severity). As a result of the generally high-risk scores of falls in our cohort, a total of 69% of the patients had documented fall-preventive measures in place prior to their fall. However, despite preventive efforts, many of these vulnerable patients continue to fall, as in our cohort, and thus are at risk of sustaining severe injuries, such as fractures or hemorrhages. Therefore, it seems prudent to complement fall prevention measures with strategies to enhance internal resistance to injuries from falls. One approach is to focus on anti-osteoporotic treatment to fortify patients who are likely to fall again despite fall preventive interventions. This is highlighted in several fall-prevention guidelines, but can still be missed in the clinical setting [11].

Our study has several limitations. Firstly, the sample size is relatively small (*n* = 159), which diminishes the statistical power and increases the risk of clinically significant differences being statistically non-significant. Additionally, the retrospective observational study design may introduce research biases, such as inaccuracies in the documentation of falls and their consequences, as well as in the assessment of risk scores. Furthermore, the study was conducted in a single ward, which may limit the generalizability of the findings. However, our study also has notable strengths. The access to a unified electronic medical record system allowed for the identification of ICD-codes and prescriptions registered across all the hospitals and primary health care centers, thereby enhancing the comprehensiveness of the data. Furthermore, there were well-implemented routines for fall risk assessment, ensuring that assessments were systematically conducted in most patients by experienced personnel.

In conclusion, although risk assessments and interventions are conducted to prevent injurious falls, a significant proportion of patients continue to experience falls. Despite being classified as high-risk fallers and having a history of recent fractures, only a minority of risk-patients received anti-osteoporotic treatment. This shortfall could potentially contribute to the high incidence of fractures resulting from falls observed in this study.

## Data Availability

The datasets generated and analyzed during the current study are not publicly available but are available from the corresponding author on reasonable request.
